# Concurrent Beet Juice and Carbohydrate Ingestion: Influence on Glucose Tolerance in Obese and Nonobese Adults

**DOI:** 10.1155/2017/6436783

**Published:** 2017-01-19

**Authors:** Joseph W. Beals, Scott E. Binns, Janelle L. Davis, Gregory R. Giordano, Anna L. Klochak, Hunter L. Paris, Melani M. Schweder, Garrett L. Peltonen, Rebecca L. Scalzo, Christopher Bell

**Affiliations:** Department of Health and Exercise Science, Colorado State University, Fort Collins, CO 80523-1582, USA

## Abstract

Insulin resistance and obesity are characterized by low nitric oxide (NO) bioavailability. Insulin sensitivity is improved with stimulation of NO generating pathways. Consumption of dietary nitrate (NO_3_^−^) increases NO formation, via NO_3_^−^ reduction to nitrite (NO_2_^−^) by oral bacteria. We hypothesized that acute dietary nitrate (beet juice) ingestion improves insulin sensitivity in obese but not in nonobese adults. 12 nonobese (body mass index: 26.3 ± 0.8 kg/m^2^ (mean ± SE)) and 10 obese adults (34.0 ± 0.8 kg/m^2^) ingested beet juice, supplemented with 25 g of glucose (carbohydrate load: 75 g), with and without prior use of antibacterial mouthwash to inhibit NO_3_^−^ reduction to NO_2_^−^. Blood glucose concentrations after beet juice and glucose ingestion were greater in obese compared with nonobese adults at 60 and 90 minutes (*P* = 0.004). Insulin sensitivity, as represented by the Matsuda Index (where higher values reflect greater insulin sensitivity), was lower in obese compared with nonobese adults (*P* = 0.009). Antibacterial mouthwash rinsing decreased insulin sensitivity in obese (5.7 ± 0.7 versus 4.9 ± 0.6) but not in nonobese (8.1 ± 1.0 versus 8.9 ± 0.9) adults (*P* = 0.048). In conclusion, insulin sensitivity was improved in obese but not in nonobese adults following coingestion of beet juice and glucose when oral bacteria nitrate reduction was not inhibited. Obese adults* may* benefit from ingestion of healthy nitrate-rich foods during meals.

## 1. Introduction

Insulin resistance and obesity, two common comorbidities, are both characterized by low nitric oxide (NO) bioavailability [1–3]. It is likely that this low NO bioavailability contributes directly to insulin resistance [2, 4], potentially via disrupted lipid handling, increased fat mass, and/or decreased glucose delivery [1, 4, 5]. For example, compared with healthy wild-type mice, mice lacking the gene for endothelial nitric oxide synthase (eNOS) develop insulin resistance and hypertension [6]. In rats, disruption of NO generation via nitric oxide synthase inhibition attenuates insulin mediated glucose uptake [7]. In humans, the D298 and IVS18 + 27 C alleles of the eNOS gene are more frequently observed in adults with type 2 diabetes compared with healthy controls [8, 9]. In light of these observations, increasing NO bioavailability may be an effective strategy to increase insulin sensitivity [4]. In this regard, ingestion of dietary nitrate may hold promise.

During the previous decade, the remarkable effects of dietary nitrate, as beet (root) juice, have been described; these effects include decreased oxygen cost of standardized physical activity [10], increased exercise performance and fatigue resistance [11, 12], and improved regulation of blood flow [13, 14]. Considerably less attention has been given to the potential influence of dietary nitrate supplementation on insulin resistance and metabolic syndrome. In rodent models of obesity/diabetes, dietary nitrate supplementation improved insulin signaling and promoted glucose transporter 4 translocation [15, 16]. Further, in eNOS-deficient mice, 10 weeks of dietary nitrate supplementation normalized blood glucose tolerance and glycosylated hemoglobin [17]. Compared with animal studies, data from human studies are sparse and do not consistently demonstrate a favorable benefit of dietary nitrate as it pertains to glucose control [18–23]. Potential reasons for these inconsistent and/or negative outcomes include issues pertaining to nitrate dosing and the short half-lives of NO and circulating nitrate/nitrite following ingestion.

The purpose of the study described herein was to determine the influence of coingestion of dietary nitrate (beet juice: BJ) with glucose on glucose tolerance in obese adults, a population with presumably low NO bioavailability [1, 3, 24], and a healthy group of nonobese adults. Dietary nitrate supplementation via BJ ingestion is thought to increase NO bioavailability through a serious of reactions: nitrate (NO_3_^−^) is reduced to nitrite (NO_2_^−^) by commensal bacteria in the oral cavity; nitrite is then converted to NO through interaction with a variety of substances in the gut and systemic circulation, including deoxygenated hemoglobin, xanthine oxidase, polyphenols, and ascorbic acid [25, 26]. The reduction of nitrate to nitrite can be inhibited with prior use of antibacterial mouthwash [23, 27–29], thus abrogating the NO-mediated beneficial effects of BJ consumption and serving as an experiment control. Accordingly, we hypothesized, in obese adults, oral glucose tolerance would be superior following coingestion of BJ plus glucose (BJ + Gluc) without prior use of antibacterial mouthwash, compared with ingestion of BJ + Gluc following prior mouthwash use. Additionally, we hypothesized that use of mouthwash would not influence oral glucose tolerance in nonobese adults following BJ + Gluc ingestion. As an additional experimental control, to determine the independent effects of mouthwash on glucose tolerance, water plus glucose was ingested with and without prior mouthwash use.

## 2. Methods

### 2.1. Research Participants

Adult members of the university campus and local community were invited to participate in the study. Inclusion criteria included the following: age within the range 18–70 years, sedentary lifestyle (≤20 min/day of exercise, ≤2 days/week), and weight stability (±2 kg) during the previous 6 months. Exclusion criteria included the following: current or past use (previous 2 years) of tobacco products, pregnancy or nursing, use of vitamins, supplements or medications known to influence glucose tolerance, history of diabetes, and concurrent participation in another study. Routine use of mouthwash was not an exclusion criterion. Participants were classified as obese when their body mass index (BMI) was ≥30 kg/m^2^. The Institutional Review Board at Colorado State University approved the experimental protocol. The nature, purpose, and risks of the study were explained to each research participant before written informed consent was obtained.

### 2.2. Experimental Design

**Figure 1:**
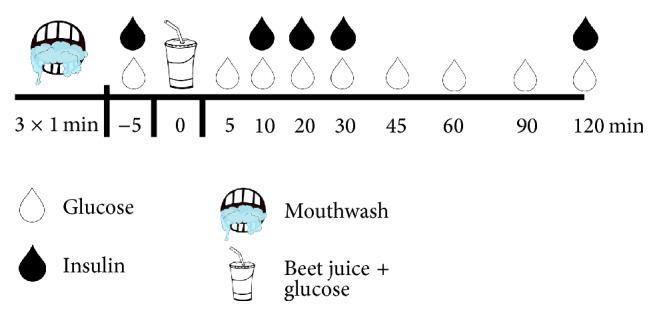
Schematic of experimental visits. Two modified oral glucose tolerance tests were administered, on two separate occasions, in a random order. At the start of one visit, participants completed 3 × 1 min mouthwash regimen. Without delay, they then ingested beet juice supplemented with glucose within 5 minutes. Venous blood (~3–10 mL) was sampled repeatedly over 120 minutes and analyzed for concentrations of glucose and insulin. See text for more details.

Two modified oral glucose tolerance tests were administered, on two separate occasions, in a random order, separated by a minimum of 7 and a maximum of 28 days (dictated by research participant availability). A schematic of the events is presented in Figure 1. Participants reported to the laboratory early in the morning following 12-hour fast and 24-hour abstention from vigorous physical activity. In addition, participants were informed as to the types of foods known to be rich in nitrates, such as leafy green vegetables, beets, and cured meats and instructed to abstain from these foods during the 24 hours prior to each laboratory visit. No additional attempt was made to control, record, or reproduce eating behavior. To prevent inadvertent removal of commensal bacteria in the oral cavity, participants also abstained from teeth cleaning, flossing, and use of mouthwash during the 18 hours prior to each test. Within five minutes, participants ingested 500 mL of BJ (Biotta, Carmel, Indiana, USA), estimated to contain approximately 17 mmol of nitrate and supplemented with 25 g of glucose (total carbohydrate load: 75 g), with and without a prior antibacterial mouthwash procedure: one 60 s rinse with 10 mL of 1.5% H_2_O_2_ (Peroxyl; Colgate Oral Pharmaceuticals, Inc., New York, NY) followed by two 60 s rinses with 10 mL of antibacterial mouthwash (chlorhexidine digluconate; Corsodyl, BCM Ltd., Nottingham, UK). Venous blood (~3 mL) was sampled immediately prior to BJ + Gluc ingestion and again after 5, 10, 20, 30, 45, 60, 90, and 120 minutes and analyzed immediately for concentrations of glucose (automated device: 2300 Stat Plus, Yellow Springs Instruments, Yellow Springs, Ohio). Venous blood (~7 mL) was also sampled immediately prior to BJ + Gluc ingestion and again after 10, 20, 30, and 120 minutes and analyzed for insulin concentrations (enzyme-linked immunosorbent assay, ALPCO Diagnostics, Salem, NH). Insulin sensitivity was estimated via the Matsuda Index [30]. If insulin sensitivity was greater without prior mouthwash, one possible explanation was that mouthwash blunted glucose tolerance. To explore this potential confounding issue, during two additional/separate visits, a subset of participants who completed the BJ experiments ingested water, supplemented with 75 g of glucose, with and without prior mouthwash use.

### 2.3. Statistical Analysis

Analysis of variance (ANOVA), with repeated measures when appropriate, was used to compare insulin sensitivity and circulating glucose and insulin concentrations in nonobese and obese adults. To determine the independent effects of mouthwash on glucose tolerance, ANOVA was also used to compare circulating glucose and insulin concentrations and insulin sensitivity. Multiple comparisons of factor means were performed using Newman-Keuls test. The level of statistical significance was set at *P* < 0.05. Data are reported as mean ± SE.

## 3. Results

**Figure 2:**
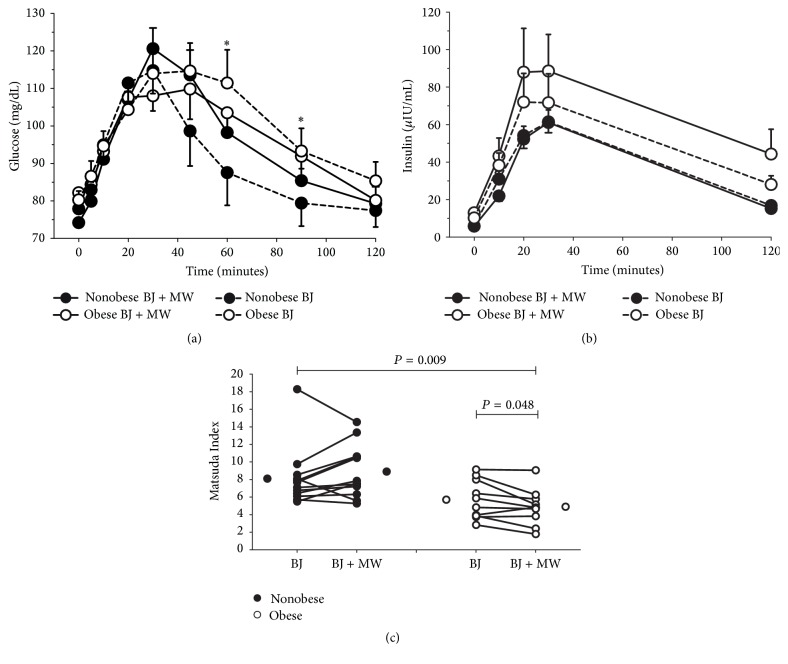
Blood glucose concentration after beet juice plus glucose consumption was greater in the obese compared with the nonobese adults at 60 and 90 minutes (*P* = 0.004 and denoted by *∗*). Inhibition of oral bacteria nitrate reductase activity with mouthwash did not influence blood glucose or insulin in either group (*P* > 0.08). Insulin sensitivity, as represented by the Matsuda Index (where a higher value is reflective of greater insulin sensitivity), was lower in obese adults compared with nonobese adults (*P* = 0.009). Inhibition of oral bacteria nitrate reductase activity with mouthwash decreased insulin sensitivity in obese adults but not in nonobese adults (*P* = 0.048). Glucose and insulin: data are mean and standard error. Matsuda Index: lines represent individual responses; stand-alone circles represent mean values.

12 nonobese (6 males and 6 females; BMI: 26.3 ± 0.8 kg/m^2^; age: 25 ± 3 years) and 10 obese adults (2 males and 8 females; BMI: 34.0 ± 0.8 kg/m^2^, age: 43 ± 4 years) were tested. Blood glucose and plasma insulin concentrations, together with insulin sensitivity, are displayed in Figure 2. Blood glucose concentration after BJ + Gluc consumption was greater in obese compared with nonobese adults at 60 and 90 minutes (*P* = 0.004). Inhibition of oral bacteria nitrate reductase activity with mouthwash did not influence glucose or insulin in either group (*P* > 0.08). Insulin sensitivity, as represented by the Matsuda Index (where a higher value reflects a greater insulin sensitivity), was lower in obese compared with nonobese adults (*P* = 0.009). Antibacterial mouthwash rinsing decreased insulin sensitivity in obese but not in nonobese adults (*P* = 0.048). Although the current study was not designed with the intention of making meaningful sex comparisons, three-way analysis of variance of the Matsuda Index data revealed no main effect of sex (*P* = 0.916) and no interaction between sex and obesity status (*P* = 0.882), sex and beet juice with/without mouthwash (*P* = 0.965), and sex, obesity status, and beet juice with/without mouthwash (*P* = 0.581).

**Figure 3:**
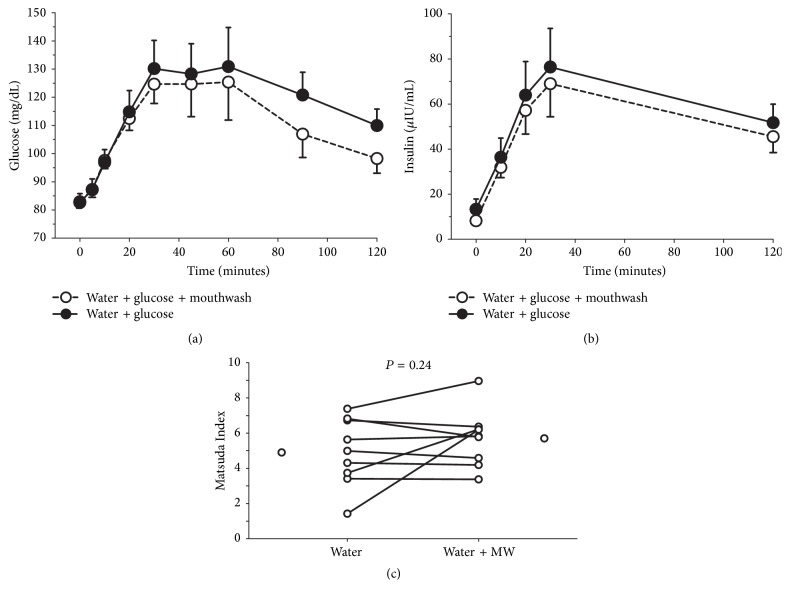
Inhibition of oral bacteria nitrate reductase activity with mouthwash did not affect blood glucose or insulin concentrations following consumption of water plus glucose (*P* > 0.83); insulin sensitivity, as represented by the Matsuda Index, was unaffected (*P* = 0.24). Glucose and insulin: data are mean and standard error. Matsuda Index: lines represent individual responses; stand-alone circles represent mean values.

In a subset comprising both nonobese (*n* = 3) and obese (*n* = 6) adults (2 males and 7 females; BMI: 33.7 ± 0.9 kg/m^2^; age: 45 ± 4 years), mouthwash did not affect blood glucose or insulin concentrations following consumption of water and glucose (Figure 3; *P* > 0.83); similarly, insulin sensitivity was unaffected (*P* = 0.24). Visual inspection did not reveal any obvious obesity-mediated differences within the water/mouthwash data.

## 4. Discussion

The novel findings of this study were the following: (1) insulin sensitivity was improved in obese but not in nonobese adults following ingestion of BJ + Gluc when oral bacteria nitrate reduction was not inhibited and (2) inhibition of oral bacteria nitrate reduction prior to ingestion of water plus glucose did not affect insulin sensitivity. The implication is obese adults at risk of developing insulin resistance* may* benefit from ingestion of healthy nitrate-rich foods during meals.

The current study is not the first to investigate the influence of dietary nitrate on glucose regulation in adult humans [18–23, 31]. Using a variety of techniques, including the hyperinsulinemic euglycemic clamp and the oral glucose tolerance test, none of the previous studies report a favorable effect of either acute or short-term (2 weeks) dietary nitrate glucose regulation [18–23]. One critical and discriminatory difference between these studies and the present study pertains to the timing of nitrate ingestion. Many of the techniques for assessing glucose regulation require that the research participant be in a fasted state, making acute delivery of dietary nitrate problematic. Accordingly, in most of the prior studies, nitrate ingestion occurred between 2.5 and 12 hours prior to assessment of glucose control; however in the present study the nitrate ingestion (as beet juice) occurred concurrently with assessment, that is, the BJ composed part of the carbohydrate challenge. Thus, the inference may be that, for maximum benefit to glucose control, obese adults should include dietary nitrate(s) (e.g., beets and leafy green vegetables) as part of their meal.

Dietary nitrate, in the absence of antibacterial mouthwash use, improved insulin sensitivity during a glucose challenge in obese but not in nonobese adults. This observation is consistent with prior reports of lower NO bioavailability in obese compared with nonobese adults [3, 24], increased NO bioavailability following BJ ingestion [25, 26], and improved insulin sensitivity following stimulation of NO generating pathways and/or dietary nitrate administration in animal models [15, 16]. Lower NO bioavailability in obese adults may be attributed to one or more factors, including increased oxidative stress [32, 33], chronic inflammation [34, 35], and/or limited availability of NO substrates such as tetrahydrobiopterin (BH_4_), citrulline, and/or L-arginine [3, 24, 36]. In turn, the lower NO bioavailability of obese adults may contribute to impaired glucose regulation on account of the important role of NO in pancreatic beta-cell function [37] and insulin dependent and independent mechanisms of glucose clearance [38, 39]. Accordingly, the potential for favorable outcomes for glucose regulation in obese adults during increased NO bioavailability, mediated by dietary nitrate ingestion, is intuitively appealing.

There are several experimental considerations worthy of discussion. For example, as described within Methods, aside from instructions to abstain from high nitrate foodstuffs prior to glucose tolerance assessment, no attempt was made to control, record, or reproduce eating behavior. While it is possible that this lack of control may have somehow biased our data and influenced our final interpretation, we feel that this is unlikely on account of the following: (1) the order of oral glucose tolerance tests with/without mouthwash was randomized and (2) there is no reason to suspect that nonobese subjects would change their eating behavior midway through the study in a manner that was different to how the obese subjects might change their behavior.

In the context of an exploratory study, we believe our data are sufficiently provocative to provide impetus for further investigation. The rigor of subsequent studies could be improved through a variety of modifications, including the addition of quantification of NO, circulating concentrations of nitrate and nitrite, and nitrate reductase activity of commensal bacteria. Inclusion of these measurements would confirm the influence of antibacterial mouthwash on the inhibition of nitrite and NO production from nitrate. Further, insight into basal differences between obese and nonobese adults pertaining to NO bioavailability and concentrations of nitrate and nitrite would also be provided. An additional consideration is the use of nitrate-free BJ, as previously described [40]. This would alleviate the need for the complicated mouthwash procedure utilized in the current study and also make the additional studies of sweetened water with and without mouthwash redundant. In the current study, the estimated nitrate content of the 500 mL of BJ was 17 mmol. Relative to previous studies, this represents a large volume of BJ and a high nitrate load and may account for some of the reported differences pertinent to glucose control [18–22]. It might be of value for follow-up studies to explore the potential of a dose-response relationship and begin to identify an optimum nitrate dose. Further, as an alternative to BJ, nitrate-rich fruits and vegetables (e.g., spinach and celery) could also be studied. Finally, from a public health perspective, we infer that obese adults at risk of developing insulin resistance may benefit from ingestion of healthy nitrate-rich foods during meals. In the current study BJ was coingested with glucose only. To support/refute our inference, follow-up studies could examine the influence of BJ coingestion with an actual, mixed-substrate meal.

## 5. Conclusions

In summary, we are the first to demonstrate that the metabolic response to beet juice combined with glucose is more favorable in obese adults when nitrate reductase activity is not inhibited. The inference is that beet juice ingestion augmented NO bioavailability and promoted insulin sensitivity. The implication for public health is postprandial glucose control in obese adults* may* be improved if dietary nitrate(s) are included as part of their meal.
